# Performance of the National Tuberculosis Control Program in the post conflict Liberia

**DOI:** 10.1371/journal.pone.0199474

**Published:** 2018-06-25

**Authors:** Kassaye Tekie Desta, T. E. Masango, Zerish Zethu Nkosi

**Affiliations:** Department of Health Studies, University of South Africa, Pretoria, South Africa; University of Cape Town, SOUTH AFRICA

## Abstract

**Background:**

Tuberculosis is a major public health problem in Liberia. According to the World Health Organization (WHO), the incidence of tuberculosis in Liberia is significantly increasing from year to year. However, little is known about the performance of the programme and the challenges after the 14 years of civil war which ended in 2003.The purpose of the study was to evaluate the performance of the TB programme of Liberia.

**Methods:**

The study utilised mixed research design; both quantitative and qualitative methods were used in this study conducted from 2013 to 2014. For the quantitative part of the study, a questionnaire, laboratory performance and eleven years TB programme data (2003–2013) were used. For the performance of tuberculosis laboratory testing, all the 107 functional tuberculosis microscopy centers in Liberia were included. For the qualitative part of the study, an interview of 10 informants and two focus group discussions (FGDs) were also conducted, each comprising of eight people. Themes and subthemes emerged from the two FGDs. Data was analysed in line with the Donabedian model. Quantitative findings were analysed and presented using both descriptive and inferential statistics.

**Results:**

The study findings pointed out that there was overall improvement in the performance of the tuberculosis control programme in Liberia from 2003 to 2013. The percentage of cured patients was 60% in 2005 and 62% in 2013. Percentage of treatment completed was 16% in 2005 and 21% in 2013. The case detection rate was 57% and treatment success rate 80% in 2013. The default rate was 11% in 2013. Of the 139 participants, 120 (86%) completed TB treatment while 19 (14%) did not.

**Conclusion:**

Between 2003 and 2013, the National Leprosy and Tuberculosis Control Programme (NLTCP) succeeded in restoring the TB services and improving some of the TB treatment outcomes including the Directly observed treatment short courses(DOTS) coverage. Despite these improvements, the TB treatment, laboratory services and human resource capacity lagged behind. The TB programme of Liberia needs to develop new strategies to address its challenges.

## Introduction

TB is a disease of antiquity, caused by Mycobacterium tuberculosis (MTB), which mainly affects the lungs. Patients with pulmonary TB whose sputum is smear-positive for M. tuberculosis form the main source of infection in communities [1]. Tuberculosis is a major contributor to the global burden of disease and has received considerable attention in the recent years, particularly in developing countries where it is closely associated with Acquired Immune Deficiency Syndrome (AIDS). About 5–10% of those without Human Immunodeficiency Virus (HIV), infected with tuberculosis, develop active disease during their lifetimes. Extra-pulmonary TB (EPTB) occurs when tuberculosis develops outside of the lungs. EPTB may coexist with pulmonary TB as well. General signs and symptoms include fever, chills), night sweats, loss of appetite, weight loss, and fatigue) and significant finger clubbing may also occur [2]. According to Global Tuberculosis report of WHO [3], there were almost 9 million new cases in 2011 and 1.4 million TB deaths (990 000 among HIV negative people and 430 000 HIV-associated TB deaths). This is despite the availability of treatment that will cure most cases of TB. Short-course regimens of first-line drugs that can cure around 90% of cases have been available since the 1980s.This shows how tuberculosis became the major global public health concern.

Diagnosis of active TB relies on radiology (commonly chest X-rays), as well as microscopic examination and microbiological culture of body fluids. Diagnosis of latent TB relies on the tuberculin skin test and/or blood tests. Diagnosis of TB in resource-poor countries is largely based on sputum-smear microscopy and chest radiography, although these methods lack sensitivity or specificity, especially when used on HIV-infected patients. Effective treatment has existed for 40 years but TB-attributable mortality remains high among HIV-infected patients in Africa, who are also particularly likely to develop TB again after receiving drug treatment for the disease [1]. TB treatment requires administration of multiple antibiotics over a long period of time. Social contacts are also screened and treated if necessary. Prevention relies on screening programmes and vaccination with the Bacillus Calmette-Guérin vaccine [4]

Antibiotic resistance is a growing problem in multiple drug-resistant tuberculosis (MDR-TB) infections. Drug resistance to M tuberculosis makes the treatment of TB relatively ineffective. The approach to TB control that is now internationally recommended is the DOTS strategy, which aims to prevent the transmission of M tuberculosis, and the related illness and death, by using combinations of anti-TB drugs to treat patients with the active disease. Unfortunately, countries in Sub-Saharan Africa are falling short of the WHO's targets for case detection and treatment. This failure is, in turn, making less likely the achievement of the Millennium Development Goals for TB to ensure that the incidence of TB is falling by 2015 and to halve the prevalence of TB and the annual number of TB-attributable deaths between 1990 and 2015. To improve the performance and impact of TB-control programmes, in the face of HIV co-infection and other constraints on DOTS, WHO has launched the revised 'Stop TB Strategy'. The new strategy, to be implemented via the Global Plan to Stop TB (2006–2015), includes intensified TB case finding, treatment of latent TB infection with isoniazid, prevention of HIV infection, cotrimoxazole preventive therapy, and antiretroviral therapy [5].

The NLTCP of Liberia functioned well prior to the 1989 civil war, but unfortunately, the Liberian civil war destroyed the health system, including the NLTCP. Primary health care infrastructure was destroyed, key personnel were killed or migrated to safety, and financial and other technical support came to a grinding halt. With the cessation of the civil war and financial support from the Global Fund, the programme structure has been revived and is on its way to full recovery [6].

## Research purpose

The purpose of the study was to evaluate the performance of the NLTCP of Liberia guided by Donabedian’s model [7] in designing strategies to improve the TB treatment outcomes for the individual TB patients, their families, health care services and the country as a whole.

## Research objectives

The research has the following objectives

- Explore the performance of the TB programme of Liberia using the structure-process-outcome model of Donabedian’s.
- Explore the performance of the TB programme of Liberia as compared to the programme objectives targets and Stop TB Partnership targets.
- Analyse the demographic, socio-economic and medicine related factors that determine the performance of TB programme of Liberia from patients and stake holders’ perspectives.

## Methods and materials

### Design

In this study, mixed sequential explanatory design was used. Both quantitative and qualitative methods were used in this study. The quantitative research paradigm was used for the National TB programme performance evaluation of Liberia (2003–2013); laboratory programme performance evaluation and TB programme performance evaluation (patient perspective) phases of the study. Qualitative study paradigm was used for the TB programme performance evaluation (TB programme stake holders and staff perspective) phases of the study. All the information obtained from the patients, staff, stakeholders, from the laboratory assessments and the records of the NLTCP of Liberia were captured and populated in to the structure, process and outcome of health care quality model. The overall performance of the programme was evaluated using the structure-process-outcome model of Donabedian.

### Settings

The study setting includes the NLTCP of Liberia and TB Annex hospital of Liberia where the TB program performance evaluation data was obtained, and the interview questionnaires were filled. For the performance of tuberculosis laboratory testing, all the 107-functional tuberculosis microscopy centers in Liberia were included.

### Study participants

The study participants included all TB patients of age 18 years and above who visited and followed up TB treatment for more than 2 months at TB Annex hospital in Monrovia. This also included patients who were referred to TB Annex from other facilities. Treatment defaulters who returned to get service during the above specified time frame were also included in the study. For the qualitative part of the study, two FGDs were conducted each composed of eight people from TB Annex hospital and eight county diagnostic supervisors from eight county hospitals. Besides, an interview of ten informants from NLTP of Liberia and different stake holders were included in the study.

### Sampling method

The sample size for the quantitative part of the study was determined using single population proportion formula. Based on these inclusion criteria, a total of 1016 patient were identified as a total population. The TB Annex Hospital TB register was used to establish the sampling frame. The register has records of the registered patient’s name, registration number, date of starting and stopping treatment, treatment outcome, HIV status, demographic details and address and classification of diagnosis. For this part of the study, simple random sampling method was used. A study conducted at TB Annex in 2011 indicated that the TB prevalence among the patients who visited TB Annex hospital was 90% [8]. The estimated prevalence of 0.9 was taken from this study. Therefore; using 95% confidence level, the sample size required was 139. These were sampled from the 1016 patients. For other quantitative study on the performance of tuberculosis laboratory testing in Liberia; all the 107 functional tuberculosis microscopy centres in Liberia were included in the study. Purposive sampling was employed and these laboratories which were accessible and with reagents available at the time of the study.

For the qualitative part of the study, purposive sampling of two focal group discussions were conducted each composed of eight participants. In addition to the focus group discussions, interview of TB program stake holders was also conducted using purposive sampling method. A total of 10 participants were interviewed.

Purposive sampling was employed in selecting study participants for the interview. In this case purposive stakeholder sampling, implies selecting those participants that are best able to answer the question at hand. Sampling for this study followed the data saturation principle in grounded theory [9], which means that more data was collected as analysis progressed, and when no new themes seem to emerge from interviews, data collection was stopped. Transcripts of discussions were undertaken from the manual notes. These final transcripts were considered as the data for analysis of the discussions.

### Data collection

The data collection for the study was guided by mixed sequential explanatory design where the quantitative data was collected followed by the qualitative data. The researcher identified specific quantitative results that call for additional explanation and used these results to guide the development of the qualitative data collection. For the study phase on evaluation of TB programme performance from patients’ perspective, data were collected by means of a questionnaire administered by registered nurses in a structured interview with each respondent. Data for laboratory performance and NLTP performance evaluation were collected by the principal investigator using self-developed and pre-tested check lists. Records and TB programme document review were used to collect the required data for the 11 years treatment outcome evaluation part of the study. The records were extracted using self- developed check list. Exhaustive literature review was done to develop the questionnaire and check lists used in this study. The questionnaire was developed in English, which is the official language of Liberia. It was piloted at the TB annex hospital and the participants in the pilot study phase were not included in the main study. The process of data collection was continued until every effort to contact every study participant in the sample had been exhausted. All completed questionnaires were picked up on daily basis from the registered nurses and were kept in a safe lockable cupboard accessible to the principal investigator only.

For the study on TB programme performance evaluation from stakeholders’ perspective, interviews of key participants were conducted after the participants gave their consent. All interviews were done in the national language (English) and took place in the participants’ offices. A semi-structured guide was used to conduct the interviews. Data collection continued until data saturation was achieved. Data saturation is a stage where new data no longer emerge during the data collection process [10].

As part of the qualitative study, two FGDs were also conducted each composed of eight people. One of the FGDs was on the performance of NLTCP as well as the TB Annex hospital as they are co-located at the same compound. The members of the focal group were randomly selected with the composition which included physicians, pharmacists, nurses, HIV-TB counsellors, administration and laboratory staff. The other focal group consisted of eight county diagnostic supervisors and the discussion was aimed at getting information about the NLTCP of Liberia laboratory performance. Participants were assured that participation or non-participation would not affect their employment or job. The two focus group discussions were guided by the principal investigator during which group members talked freely and spontaneously about the TB programme service at the TB Annex hospital and their perceptions on the performance of NLTCP. The focus group discussion was captured on FGD guide and later transcribed for analysis.

### Data analysis

Both quantitative and qualitative data analyses were used for the study. For the quantitative part of the study on patient perspective of the TB programme performance, all the questionnaires and check lists for all the study samples were collected, the researcher reviewed all of them for completeness and performed appropriate data cleaning. Data were entered in to EXCEL and imported in to Statistical Package for Social Sciences (SPSS) version 20 used for statistical analysis. Both descriptive and inferential statistics were used to describe the study subjects in relation to relevant variables. Descriptive statistics were used to summarise socio-demographic data, treatment compliance and laboratory results. Descriptive summary statistics and graphical summaries in charts (pie, bar, cross-tabulations) were used. Chi-square tests of association were conducted to assess dependence relationships among potential factors. A p-value <0.05 was considered significant [11].

For the qualitative part of the study, qualitative content analysis was conducted considering the transcripts the two FGDs and one interview. First the transcripts were read and re-read to gain familiarity with the data and a sense of the whole. Following this, each transcript was coded openly, identifying phrases, words and sentences that formed meaning units. These were then converted into condensed meaning units. These condensed meaning units were then abstracted further and labelled with a code. Codes were further arranged according to sub-themes and major themes [12].

### Ethics statement

Prior to conducting the study, permission was obtained in the form of a clearance certificate from the Higher Degree Committee of University of South Africa and National Ethics Board of Liberia. Besides; permission to conduct the study was obtained in a written letter from the National Leprosy and Tuberculosis Control Programme of Liberia. This was done as soon as ethical approval was granted by the Research and Ethics Committee of the Department of Health Studies, University of South Africa. Written consent was obtained from the study participants (S1 Text). No information was given to third party without the permission of the Ministry of Health of Liberia.

## Results

### National Leprosy and Tuberculosis Control Programme of Liberia performance from patients’ perspectives

A total of 139 study respondents were interviewed of which 120 were TB treatment compliant and 19 were non-compliant. Of the 139 respondents, 120 (86%) completed TB treatment while 19 (14%) did not. The 19 respondents who were non-compliant gave the following reasons for not completing their treatment. Six (32%) of them stopped taking the drugs thinking that they felt better; 1 (5%) discontinued treatment because their home was far away from the TB hospital. The following reasons were given for stopping the drugs; stigma 2 (11%), lack of family support 1 (5%), no food available 1 (5%), side effects of drugs 2 (11%). Besides; not feeling better on medicine 1 (5%), cost of travel 1 (5%) and medicines not working 2 (11%) were reasons given by the study respondents for not completing their TB treatment. The socio-demographic factors included age, gender, marital status, level of formal education and religion (S1 Table). The demographic factors are presented below (Table 1).

**Table 1:** Demographic factors for TB treatment compliant and non-compliant study respondents.

Demographic factors	Compliant(N = 120)		Non-compliant(N = 19)		Significance	
	Number	Frequency (%)	Number	Frequency (%)	X^2^	p
**Age group**						
18–25	18	15	4	21	66.9	<0.01
26–33	34	28	7	37		
34–41	24	20	3	16		
42–49	17	14	2	11		
50–57	8	7	2	11		
58–65	14	12	0	0		
66–73	4	3	1	5		
>73	1	0.8	0	0		
**Gender**						
Male	58	48	9	47	0.18	0.671
Female	62	52	10	53		
**Marital status**						
Single	24	20	6	32	44.2	<0.001
Married	60	50	8	42		
Divorced	19	16	2	10.5		
Widowed	17	14	3	15.5		
**Religion**						
Christian	64	53	13	68.4	74.8	<0.001
Islam	27	22.5	4	21		
Traditional	19	15.8	2	10.5		
No religion	10	8.3	0	0		
**Level of education**						
None	16	13	1	5.2	32.4	<0.001
Primary	50	42	11	57.8		
Secondary	32	26.7	5	26.3		
Tertiary	22	18.3	2	10.5		

N = number; X^2^ = Chi square statistic, p = p-value

65% of respondents were between 18 and 41 years of age. This shows that most of the respondents were in the economically productive age group. There was difference to treatment compliance at age of 58 and above between complaints and non-complaints (P<0.001). This indicates that the old age groups were more compliant to TB treatment compared to the young age group (Table 1). Christians and Muslims were more on the non-compliant side compared to the traditional and non-religious respondents who were more compliant to treatment (p<0.001). This difference might have been the result of underestimation of the value of the TB drugs by religious people by focusing on their religious practice. There was a higher proportion, 14 (54%) of those married in the compliant group compared to the non-compliant group and these differences were significant (p<0.001). This might be associated with the treatment support married couples obtain from their partners. The level of education attained was not a significant factor influencing TB treatment compliance (p = 0.7).

The following factors were assessed as patient related factors for treatment compliance; smoking, drinking alcohol and availability of treatment supporter. Smoking cigarettes in the previous six months was however not associated with any statistical difference between the compliant and non-compliant groups (p = 0.58). in contrary, there was a significant difference between those who had drunk alcohol in the previous six months and those who had not done so with respect to TB treatment compliance (p<0.001). This indicates that there were more respondents in the compliant group who didn’t drink alcohol during the six months TB treatment duration. In this study, having a treatment supporter was associated with significant difference between compliant and non-compliant groups (p<0.001). Those respondents with treatment supporters were found to be more compliant to the TB treatment compared to those who had no treatment supporter.

The socio-economic factors described include the employment status, number of dependants and availability of food during the period the patient was on TB treatment. Unemployment was a significant factor contributing to non-compliance to TB treatment (p<0.001). The difference between the compliant and non-compliant groups for availability of food was significant (p<0.001); those who got food to take with their medicine were more compliant to the treatment.

The health care structure and process related Donabedian’s factors assessed include the convenient TB facility working hours, opening time of the TB facility, the attitude of the health worker at the TB facility, distance travelled to the TB facility, convenience of the working hours at the TB facility and availability of medicines at the facility. There was no significant difference between the compliant and non-compliant groups with regards to convenient TB facility opening times (p = 0.34) and TB service hours(p = 0.006). But there was significant difference between TB treatment compliant and non-complaints with regard to distance travelled to the TB facilities and health workers’ attitude to patients (p = 0.009) and (p<0.001) respectively. To determine TB treatment default factors, patients were asked with whom they live with and the number of people in the house. There was a significant difference between the two groups with respect to who the respondent lived with (p<0.001). Those who lived alone were more non-compliant to TB treatment compared to those who lived with family members. In addition to demographic, socioeconomic and health related factors; stigma and discrimination were also assessed in this study. The disclosure of TB status was associated with significant difference between the compliant and non-compliant groups (p = 0.014). Those who disclosed their TB status were more compliant compared to those who didn’t.

Knowledge of TB disease and HIV status of patients was also assessed in this study. Out of 139 respondents 62 (45%) patients correctly identified coughing, chest pains, night sweats and loss of weight as symptoms of TB. 20 (14%) of the respondents knew the coughing and night sweat as symptom of TB. Of the 139 respondents, 130 (94%) of the respondents had an HIV test result known and 9(6%) were with unknown HIV status. Sixty (43%) of all the respondents with an HIV test result were HIV positive, 70 (51%) were HIV negative. Nine (47%) of the 19 HIV positive respondents did not complete TB treatment compared to 8 (31%) HIV negative respondents. Nine (47%) of those who didn’t complete treatment were HIV positive while 51 (42%) of those who were HIV positive completed treatment. HIV status was a significant factor contributing to compliance or non-compliance to TB treatment (p<0.001). Out of the sixty HIV positive TB patients who participated in the study, 40 (67%) of the HIV positive respondents were on anti-retroviral therapy (ART) initiated whereas 20 (33%) were not on ART initiated. Forty-six (77%) of the HIV positive respondents were given cotrimoxazole preventive therapy whereas 14 (23%) were not given a cotrimoxazole preventive therapy.

### The National Leprosy and Tuberculosis Programme of Liberia programme performance from staff’s perspectives

The NLTCP of Liberia performance was evaluated from staff’s perspective. Two focus groups were conducted. The first one was composed of 8 county diagnostic supervisors. The second one included eight staff from the TB Annex hospital. The following themes and subthemes (Table 2) were emerged from the two FGDs and discussed in detail on the following two sections.

**Table 2:** Themes and subthemes extracted from the FGD participants’ responses.

FGD participants	Themes	Subthemes
County diagnostic supervisors (n = 8)	Lack of NLTCP support	Lack of mentorship
		Dual responsibility
		Work overload
	Lack of NLTCP commitment	Poor scope of practice
		Lack of incentives
	Integrated supervision which is not TB lab focused	Poor quality of supervision
		Poor performance at TB labs
TB Annex hospital staff (n = 8)	Lack of support from NLTCP	Low salary
		Lack of incentives
		Shortage of food and TB drugs
	Poor patient education	Lack of TB knowledge
		High risk of TB infection
		Poor TB treatment adherence
	Poor staff and patient satisfaction	High staff turn over
		Low treatment success rate
		High default rate
		Poor service at the facility

The first FGDs were asked whether there was improvement or not in the laboratory performance of the National Tuberculosis programme in the last five years (2009–2013). Six FGDs (75%) explained that there was no improvement at all. On the question that addressed the opinion of the FGDs on the achievements of the TB programme in the laboratory diagnosis; 7 (89%) of the FGDs said that there was no significant achievement in the programme in the last couple of years.

The second FGDs from the TB Annex hospital were asked how the information to patient was provided in their facility. The responses were through health education and peer counselling. Most FGDs (88%) explained that information and education on TB diseases was provided as per the National TB guideline. The FGDs also responded on whether they are getting enough support from the TB programme and from the TB hospital administration to discharge their responsibility or not. Six out of the eight (75%) FGDs said no support was provided from the programme to discharge their responsibilities. FGDs were also asked about the overall performance of the National tuberculosis programme i.e. whether the programme was performing well or not. Only 2 of the 8(25%) FGDs indicated that there was improvement in different programme indicators. The two FGDs explained the improvement in outcome indicators of treatment completion and success rates. In the contrary an FGD woman argued that there was high death and default rate.

### The TB programme performance evaluation (TB programme managers and partners perspective)

The National Tuberculosis Programme managers and partners were interviewed about the tuberculosis programme performance using open ended questionnaire. On the question raised on partnership, 80% of the interview participants said that there was lack of partnership in the programme. Finally, interview participants were asked if there were any improvements made by NLTCP after the civil war that ended in 2003. All the participants indicated that few improvements were achieved in the outcome and programme performance indicators. Most of them also explained that global TB outcome targets were not met despite the continuous improvement. Getting accurate and reliable data of TB services from the 15 county facilities is a critical challenge noted by 8 (80%) of the participants as one of the challenges. The following challenge was emphasised by one of the participants. The following challenges were also indicated by the participants:

- The screening of contacts of TB patients, particularly smear positive cases remains a gap in the programme.
- The TB/HIV collaboration mechanism is not operational.
- Challenges in reporting TB data through the Health management information system (HMIS).
- MDR surveillance has never been conducted in Liberia. Prevalence study is not also conducted.
- The national burden of MDR-TB has not been established as planned in the national strategic plan 2007–2012.
- The NLTCP has not developed a national framework for community TB care that defines the referral linkages.

### National Leprosy and Tuberculosis Control Programme of Liberia laboratory testing performance

As part of the performance assessment of the TB microscopy testing sites, 80 facilities participated in the proficiency panel testing (S2 Table). TB specimens were prepared on slides at the National Reference Laboratory of Liberia and distributed for facilities to test them as part of their routine TB smear microscopy testing. Ziehl Nelson staining technique and WHO TB External quality assessment (EQA) preparation guidelines were used by the national laboratory to prepare the slides. Each slide carried 10 points and the total maximum score was 100 (for 10 slides). A performance score less than 90 (90%) was considered to be unacceptable by WHO TB EQA standard. The analysis of the results was as follows.

As it can be seen from the Table 3 above, only 20 (25%) of the participating laboratories had very good and excellent performance which was acceptable performance. Only two facility laboratories scored 100%. One of the major EQA methods for sputum microscopy is on site assessment with standard check list which includes all aspects of TB laboratory testing. It includes assessment adequate laboratory space, safety, trained staff, laboratory standard operating procedures (SOPS), laboratory equipment and supplies.

**Table 3:** Performance of proficiency panel participated TB microscopy testing sites (N = 80).

Facility performance	Number and % of laboratories	Liberia NLTCP scale	WHO recommended scale
Poor	9 (11%)	0–55	<65
Fair	8 (10%)	60–65	65–70
Fairly good	19 (24%)	70–75	70–80
Good	25 (31%)	80–85	80–85
Very good	18 (23%)	90–95	85–95
Excellent	2 (2%)	100	>95
Total number	80 (100%)		

For the phase of the study on the performance of tuberculosis laboratory testing in Liberia; 107 functional tuberculosis microscopy centres in Liberia were included in the study. But only 93 laboratories were visited and included in the assessment. In 40% of the laboratories assessed for the study, the TB sputum microscopy testing was conducted by laboratory aids that were not formally trained for laboratory science. 59% of the laboratories accessed did not have separate area for TB work and 34% didn’t have separate table for TB specimen processing. This indicates that the minimum room and space standards were not in place when the TB microscopy laboratories were constructed. The assessment findings also indicated frequent stock out of TB slides, slide boxes, staining reagents and specimen cups during the assessment year.

### Trends of tuberculosis programme performance in Liberia; documents and reports review and analysis

**Fig 1:**
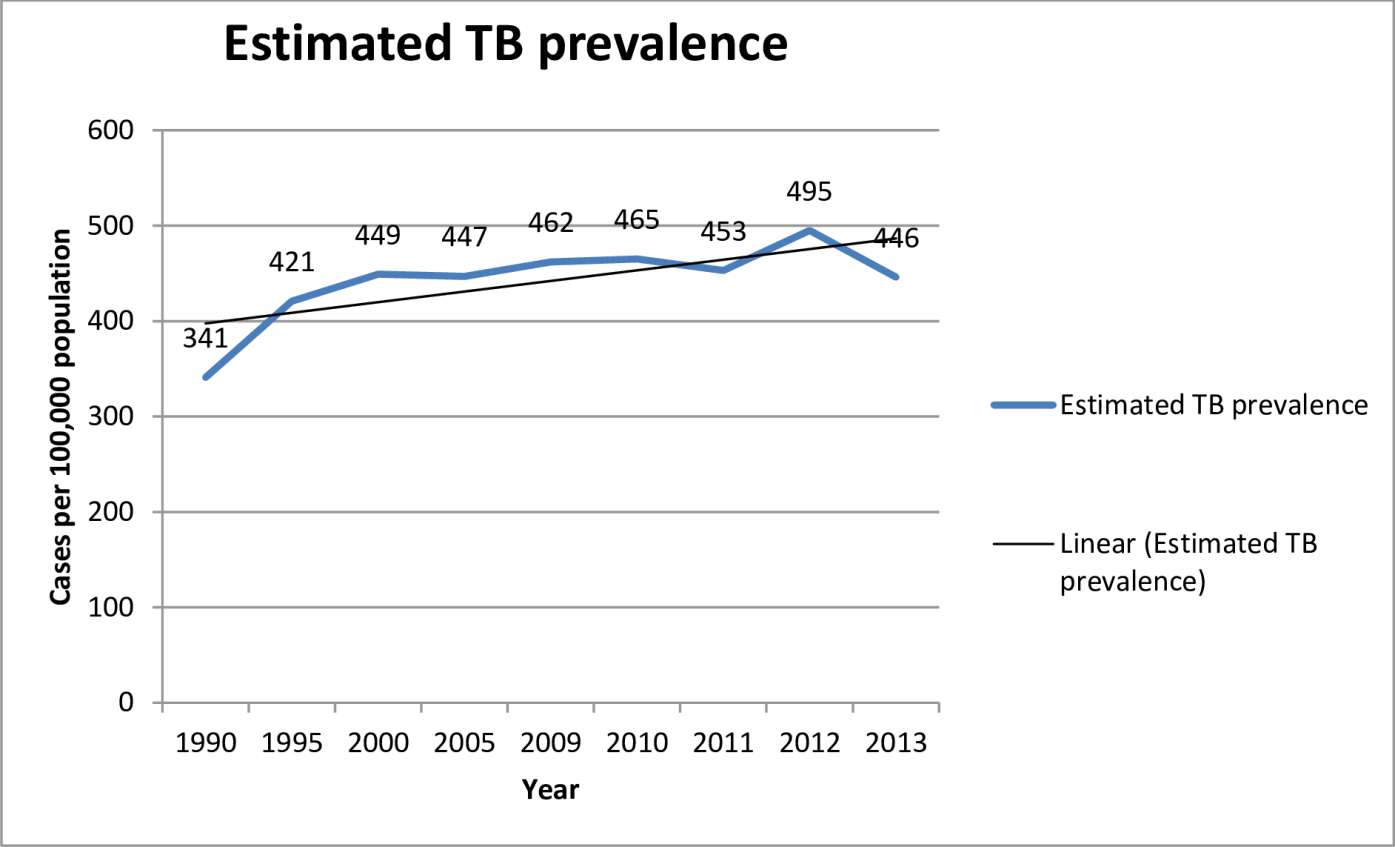
Estimated Tuberculosis prevalence (the number of cases of all TB forms in a population at a given point in time, expressed as a rate per 100 000 population) in Liberia from 1999 to 2013.

**Fig 2:**
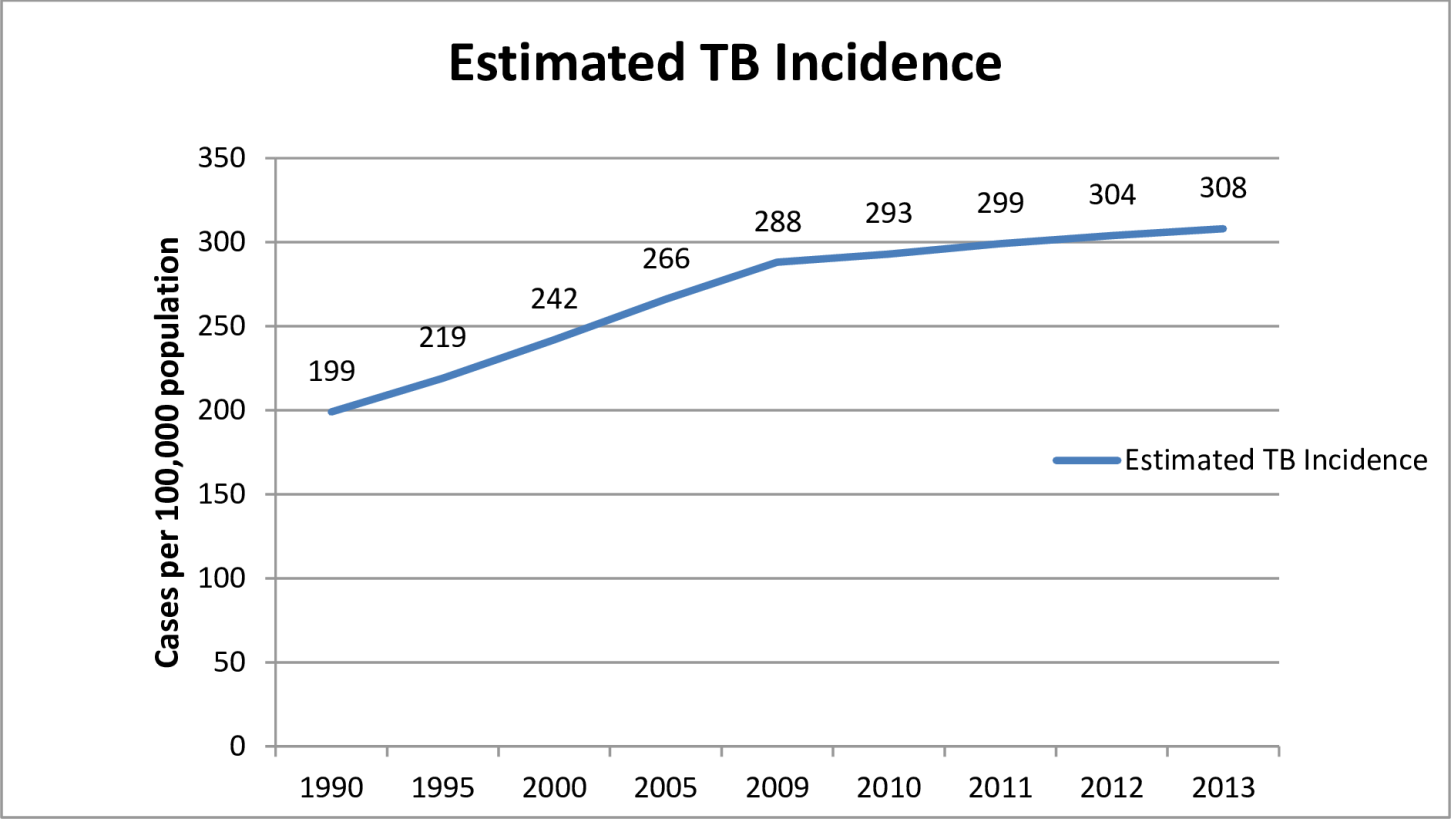
Estimated TB incidence (the number of new and relapse episodes of all forms of TB occurring in a given year) in Liberia from 1990 to 2013.

This part of the study is aimed at evaluating the eleven years (2003–2013) performance of the tuberculosis programme in Liberia (S3 Table). To achieve the objectives of the study, TB prevalence, Incidence, mortality, TB case notification and TB/HIV co-infection were analysed from data obtained from TB programme documents and the Ministry of Health of Liberia HMIS unit. The TB prevalence significantly increased from year to year with a slight decline in the year 2011 and 2013 (Fig 1. Estimated prevalence of Liberia from 1990 to 2013). The incidence of Tuberculosis in Liberia is significantly increasing from year to year (Fig 2. Estimated TB incidence of Liberia from 1990 to 2013).

### TB case notification in Liberia

**Fig 3:**
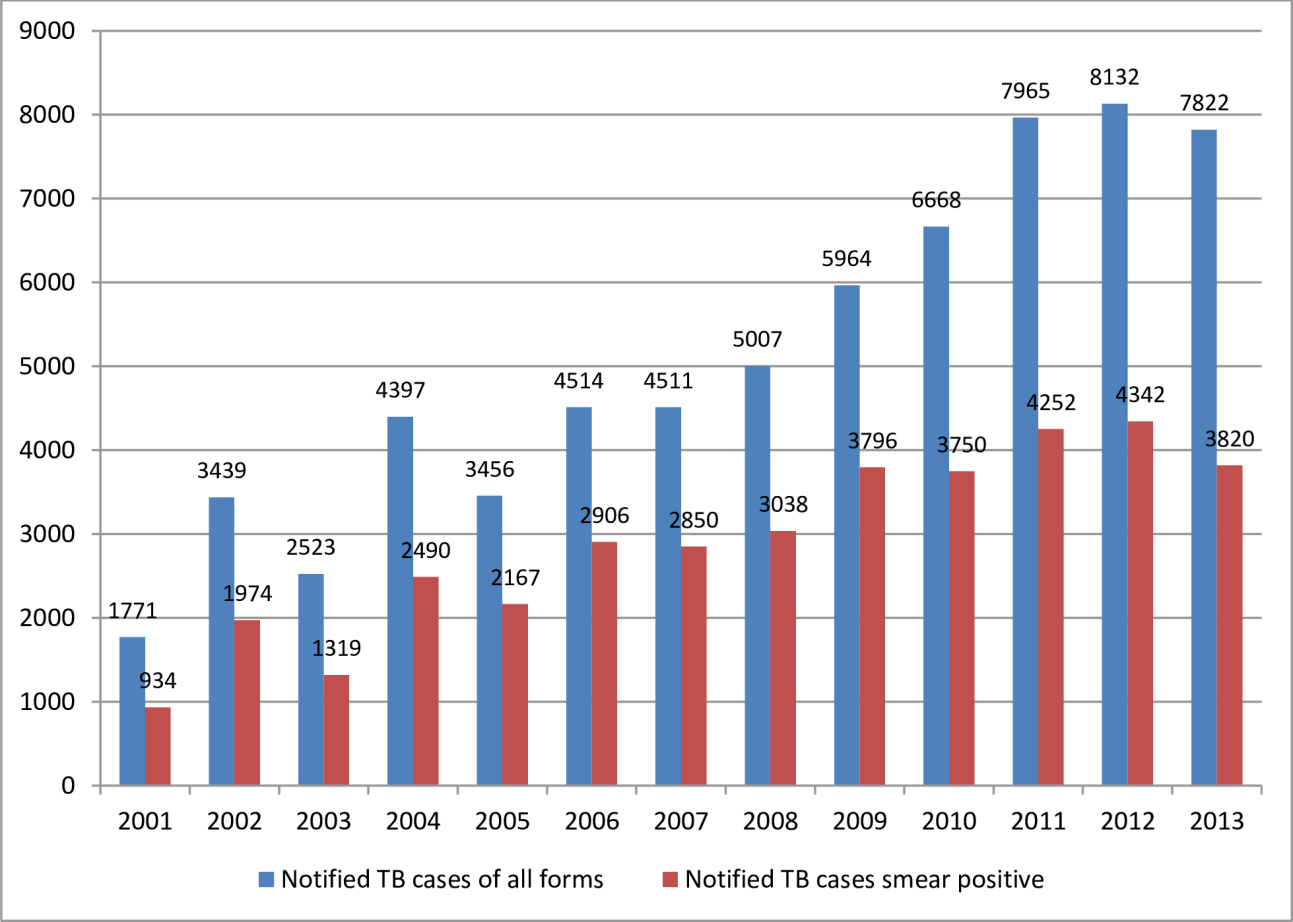
Tuberculosis case notification in Libera from 2001 to 2013. The graph in blue indicates notified TB cases of all forms and the graph in red indicates notified TB cases of smear positive per 100,000 population.

In 2013, the programme registered and notified a total of 7882 TB cases of all forms of which 3820 were pulmonary smear positive TB cases. (Fig 3. TB case notification in Liberia from 2001–2013) shows the notified TB cases of both smear positive and all TB forms. The programme has increased the numbers of all forms and smear positive notified in the last eleven years (2003–2013).

**Fig 4:**
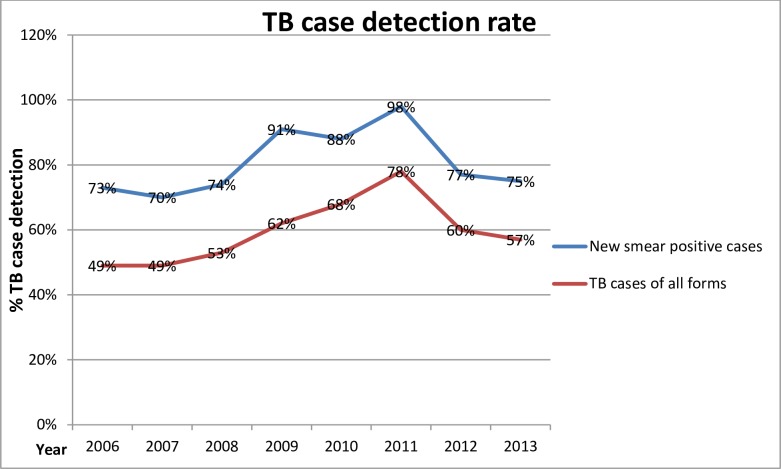
TB Case detection rate in Liberia from 2006 to 2013. The line graph in blue indicates detected TB cases of all forms and the line graph in red indicates detected TB cases of new smear positive.

The notification rate was 2523 for all TB cases and 1319 for smear positive in 2003. This shows a continuous increase in cases notified after the war ended in 2003 as indicated in the Fig 4 (TB Case detection rate in Liberia 2006–2013). The highest notification rate was obtained in 2012 where the all forms of TB notified were 8132 whereas the smear positive notified cases were 4342.

### TB/HIV co-infection trends in Liberia

According to the National HIV prevalence survey conducted in 2012, The HIV prevalence rate in the general population is estimated to be 1.9%. A sero-prevalence survey of HIV among TB patients conducted by the National AIDS Control of Liberia in 2009 revealed TB/HIV co-infection rate 22.3%.

The number and percentage of TB patients who were tested for HIV was 3272(49%) in 2010 and increased to 58599 (75%) in 2013 as indicated by the Table 4 above. Percentage of HIV positive TB patients started or continued on CPT was only 8% in 2010 and reached 74% in 2013.The ART coverage for HIV positive TB patients in Liberia was 16% in 2013, very low (Table 4).

**Table 4:** TB/HIV co-infection in Liberia (2009–2013).

TB/HIV Indicators	2010	2011	2012	2013
Notified TB cases	6668	7965	8132	7822
Number of TB patients with known HIV test result	3272	4355	5661	5899
% of HIV^+^ TB patients with known HIV test result	49%	55%	70%	75%
TB patients with positive HIV test	283	454	772	950
% of HIV ^+^ TB patients started or continued on CPT	8%	68%	42%	74%
% of HIV^+^ TB patients started or continued on ART	6%	10%	14%	16%

### Performance of the Leprosy and Tuberculosis Programme of Liberia as compared to Stop TB strategy 2006–2015

**Fig 5:**
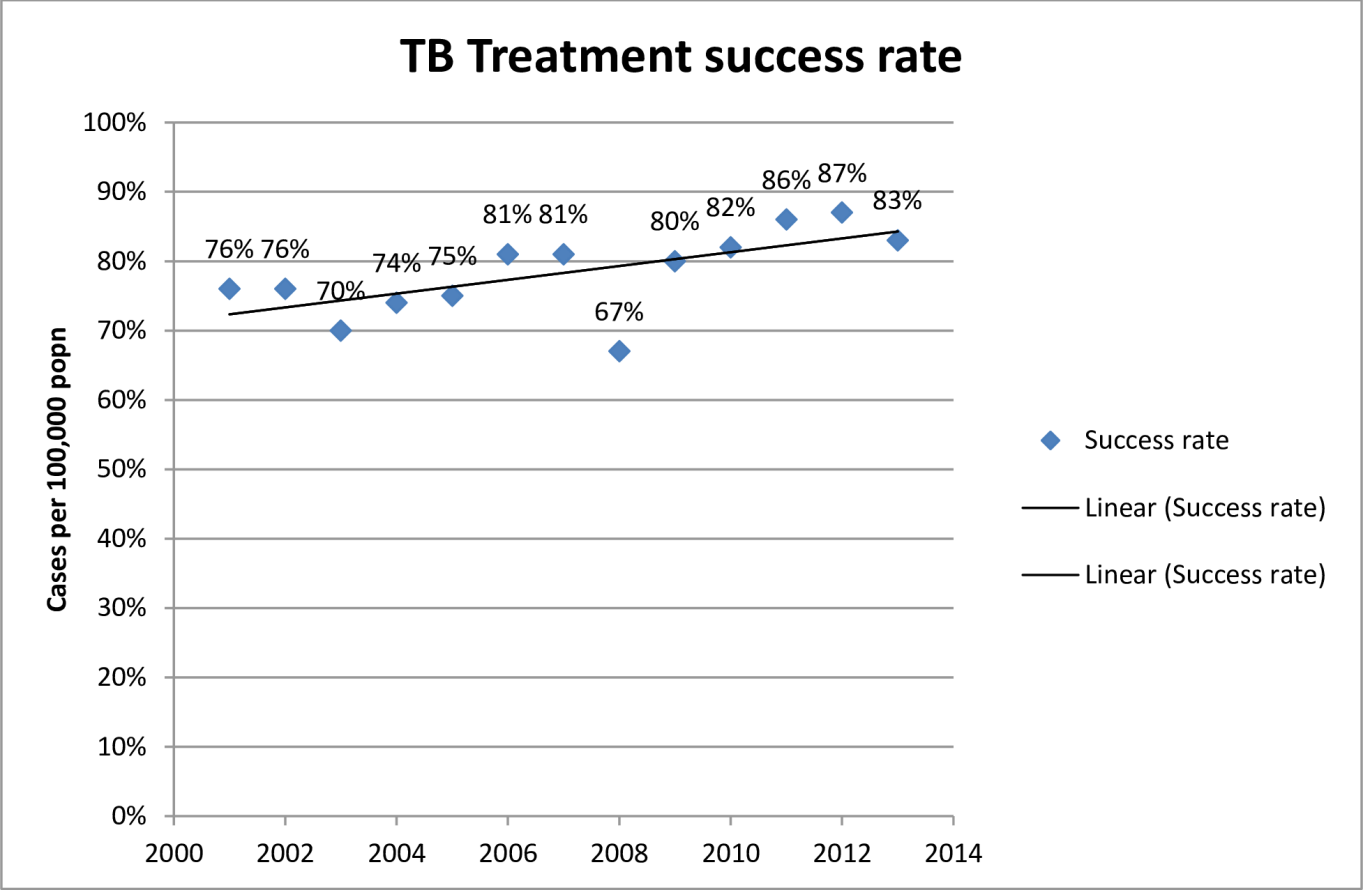
Tuberculosis treatment success rate (proportion of TB cases successfully treated; i.e. cured plus treatment completed among all TB cases notified) in Liberia from 2000 to 2013.

The TB case detection rate, treatment success rates and TB treatment cure and default rates were considered as outcome components of the Donabedian’s model and used as one aspect of evaluating the performance of NLTCP of Liberia (Fig 5. TB treatment success rate in Liberia from 2000–2013).

The TB case detection rate of all forms in Liberia was 57% in 2013. This was by far lower than the case detection target set by STOP TB target. The global target for TB control through STOP TB full DOTS expansion was the attainment of 70% case detection. Treatment success rate was also used as one of the outcome indicators for the NLTCP of Liberia using Donabedian’s model. The global target for TB control through STOP TB full DOTS expansion was the attainment of 85% treatment success rate by 2005. Liberia made progress in maintaining a high treatment success rate above 80% in 2011 and 2012. In 2013, the treatment success rate was 83% which is below the global target (Fig 5).

TB treatment outcome of cured rate, completed rate, failure rate, death rate and default rate was also used as measures of performance as components of outcome part of the Donabedian’s model. The most important cause of unfavourable treatment outcome in Liberia is the default rate which was 11% in 2013. This has nevertheless by far increased from a level of 9%, 6%, 4% in the 2010, 2011and 2012 respectively (S4 Table).

## Discussion

Tuberculosis is a major public health problem in Liberia. According to the World Health Organization report of 2014; the TB prevalence in Liberia for the year 2013 was 446/100,000 population compared to 341/100,000 in 1990. The incidence of all forms of TB was 308 per 100, 000 populations. The incidence of TB in Liberia is significantly increasing from year to year. According to WHO report of 2014, the incidence rate of guinea and Nigeria for the year 2013 was 177 and 338 per 100,000 populations respectively. This shows Liberia had higher incidence rate than the two West Africa countries. The TB mortality in Liberia for the year 2013 was estimated at 45/100,000 populations by WHO report 2014. The mortality rate was 35/100,000 populations in 1990 and reached 53/100,000 population in 2000. According to the 2013 review of TB programme of Liberia documents and the information obtained from the key interview participants of this study, the programme registered and notified a total of 7882 TB cases of all forms of which 3820 were pulmonary smear positive TB cases.

### Evaluate the performance of the TB programme using the structure-process-outcome model of Donabedian

The structure-process-outcome model of Donabedian was used to assess the performance of NLTCP of Liberia. All the information obtained from the patients, staff, stakeholders, from the laboratory assessments and the records of the NLTCP of Liberia were captured and populated in to the structure, process and outcome model of Donabedian. Structural aspects of quality were assessed based on the variables of government’s political commitment, availability of diagnostic algorisms, SOPs, infrastructures, access, laboratory safety, waste disposal, staffing, availability of drugs and patient environment. The Government of Liberia has strong commitment to TB care and control. The interview with TB programme managers and partners of this study clearly indicated that tuberculosis care and control is the highest priority in the Ministry of Health and Social Welfare of Liberia. Even though TB control is among the stated priorities in Liberia, the programme is not well funded because of limited national budget. The study part on patients’ perspectives clearly showed this gap. The major gaps identified in this study regarding financial resource are lack of sustainable domestic funding and poor resource mobilisation. A study conducted in Ghana on political commitment for TB control [13], indicated this limitation. Availability of diagnostic algorisms, SOPs, laboratory infrastructures, access, laboratory safety and waste disposal were the Donabedian’s structural aspects assessed to evaluate the performance of the TB laboratory diagnosis in Liberia. The review of documents of the TB programme indicated that TB laboratories have diagnostic algorism provided by the NLTCP of Liberia which was revised in 2009. The study findings on the laboratory performance indicated that there were no standard operating procedures; instead laboratories have job aids with limited information. Lack of laboratory equipment, waste disposal materials and dedicated space/room for TB laboratory testing were identified as major gaps in this study. In this study, only 76% of the TB laboratories assessed were well ventilated and only 41% were with separate area for TB laboratory work. Stock out of reagents, slides and slide boxes were another challenge identified during the onsite assessment of this study. Adequate slides were not available in 74 (80%) of the TB testing laboratories assessed. A study conducted on TB laboratory external quality assessment in Ghana [14], indicated that the National TB programme approved SOPs were pasted in all the laboratories for easy reference and staining reagent containers were properly labelled with the date of preparation and expiration. This is different finding from this study.

Application of national guidelines for TB case finding and diagnosis is one of the strengths of NLTCP. According to the review of TB programme report and interview with respondents of this study, the diagnostic approaches are consistent with national guidelines. Staffing levels at TB facilities and presence of trained TB care provider were also included as part of the Donabedian’s structure factor for this study. According to the interview with respondents of this study, TB human resource development which is so important for supporting effective and efficient implementation of the Stop TB Strategy was a critical gap identified in Liberia. There is lack of medical doctors, pharmacists, nurses and laboratory technologists at different level of the health system. The study finding on the TB laboratory performance showed that most of the TB microscopy testing in Liberia health centers was conducted by laboratory aids who were trained only on the job to perform TB microscopy. According to the information obtained from the key respondents of the interview and the reports of facilities; shortages of anti-TB drugs were considered the major problem in TB treatment and care in Liberia.

### Performance of Donabedian’s process aspects of TB service care in Liberia

The healthcare process related Donabedian’s factors assessed in this study included convenience of the TB facility working hours, waiting time at the TB facility, distance of the TB facility (clinic) from patients’ home, the attitudes of the health workers at the TB facility and patients’ knowledge about their TB disease. In this study, 85 (71%) of the participants replied that TB facility opening time was acceptable for them. Despite the findings of this study, The TB facility opening and working hours have been shown in many studies to be important factors affecting TB treatment compliance [15, 16, 17, 18]. In this study eighty-five (61%) of the participants indicated that distance to the clinic was not a problem for them while 54 (39%) said it was not easy to reach the TB facility. Other studies found that the further the clinic was from the patients’ home, the more chances the patient would be non-compliant [15, 16]. The other factor considered in the Donabedians process aspect was health workers attitude towards the TB patients. In this study, there was significant difference between the compliant and non-compliant groups with respect to the staff rating for attitudes to their patients. Respondents indicated that they found the attitudes and behaviours of health professionals towards them demeaning. Health workers attitudes, such as being unfriendly to patients tend to deter patients from seeking treatment or coming to collect medicines. In a similar study conducted in Ghana, most patients indicated that they found the attitudes and behaviours of health professionals towards them demeaning. They described such attitudes as affecting their confidence and the way they related to others in the community [19]. Several studies indicated similar findings [15, 16]. The other Donabedian’s TB process factor analysed in this study was patients’ knowledge about their TB disease. The patients’ knowledge of symptoms of TB was low as only 59% participants managed to identify coughing, chest pains, loss of weight and night sweats as presenting symptoms of TB. In the contrary, the knowledge of the TB treatment duration was high as 83% knew the duration of TB treatment. The findings of this study are similar with several studies [20,21,22, 23]. Lack of health education results in patients not understanding the importance of complying with treatments. Feeling better was cited among reasons for default and has similarly been reported in other studies as cause for default [22,23]. Adequate patient education and counselling at initiation of treatment is therefore important and could mitigate early default. In order to solve the problem of inadequate information, the NLTCP of Liberia has to develop a standardised approach to patient health education.

### Performance of Donabedian’s outcome aspects of TB service care in Liberia

The outcome aspect of the Donabedian model was assessed by treatment success rate, case detection rate; default rate and death rate. This was done by collecting the information from the eleven years (2003–2013) of NLTCP data review. Only the last year of the study (2013) performance was considered for the measurement of current performance. This was further compared with the yearly TB programme targets and the STOP TB WHO global target. The overall goal of the NLTCP of Liberia is in line with the Millennium Development Goals (MGD) and Stop TB Partnership targets. MDG 6, Target 8 is to halt and begin to reverse the incidence of TB by 2015. This was clearly stated in the five years (2007–2012) strategic plan of NLTCP of Liberia. The STOP TB Partnership targets expand on the MDG and one of the strategic objectives is to effectively pursue high-quality DOTS expansion and enhancement through decentralised laboratory and DOTS services [5]. According to the key respondents of the study and the review results of the documents of NLTCP of Liberia, the country has made progress in maintaining a high treatment success rate above 80% throughout the years 2009 to 2011. The average national treatment success rate for the 15 counties was 80% in 2013. This national average treatment success rate was lower than the STOP TB WHO target of treatment success rate above 85% which was set in 2005. The critical challenges mentioned by the study participants of this study were delay in the release of funding from Global fund, inefficient DOTS decentralisation to peripheral health facilities and impaired distribution strategy of TB drugs and poor data reporting mechanism. NLTCP of Liberia has to design effective intervention strategy to improve the treatment success rate and met the STOP TB target of above 85% treatment success rate.

The TB case detection rate of all forms in Liberia was 57% in 2013. This was by far lower than the case detection target set by STOP TB. The global target for TB control through STOP TB full DOTS expansion was the attainment of 70% case detection. One major constraint identified as limiting the attainment of this target was the non-involvement of the private sector in the TB control programme in Liberia. The target of 70% case detection would not be reached unless DOTS programmes continue to expand geographically as well as involve the private sector. This target is not attained by most countries in Africa which was indicated in WHO TB global report 2014. In a study conducted by Mauch, Weil, Munim, Boillot, Coninx and Sevil [24], in fragile states of Afghanistan, Democratic Congo, Haiti and Somalia; case notifications and treatment outcomes increased in all four countries since 2003 (treatment success rates 81–90%). Access to care and case detection however remained insufficient (case detection rates 39–62%) and the study finding for these four countries was similar to this study. Despite the challenges in management, coordination, security, logistics and funding, TB control programmes can function in fragile states [24].

The review of NLTCP of Liberia documents pointed out that the most important cause of unfavourable treatment outcome was default rate which was 6% in 2011 and increased to 11% in 2013. The study participants mentioned that there was no well-organised defaulter tracing mechanism in place. In a study conducted in Sudan, the TB control programme in Khartoum state achieved a default rate of 14.1% [25].

Review of the reports and the information obtained from the interview respondents demonstrated that the DOTS treatment network was expanded from 200 in 2007 to 450 by the end of 2012. Despite the significant scaling up of the DOTS service, coverage by districts was less than 90%. This shows 100% coverage was not attained in Liberia though Liberia adopted the WHO DOTS strategy in 1990. One of the main reasons for not attaining 100% DOTS coverage was the fact that community-based DOTS was not implemented. The NLTCP didn’t develop a national frame work for community TB care that defines the referral linkage. The interview with the programme managers indicated that the scale-up of community-based interventions was impaired by delay in meeting the requirements of the global fund support with respect to finalisation of the mapping the general community health volunteers in all counties and the delay in identification of supervisors at the health facility level.

The other two areas assessed in this study in relation treatment outcome are to case detection MDR TB and HIV/TB status in Liberia. The NLTCP of Liberia developed strategy to assess the status of MDR-TB in the country in 2010. The strategic plan indicated that the TB programme was to start treating MDR cases in 2011 with full capacity for MDR laboratory testing. Since the development of the strategy in 2007, very limited activities were executed to address the world-wide challenge of MDR TB. TB drug resistance survey was not conducted, and the burden of MDR-TB in Liberia is not yet determined. The interview with the study participants indicated that the programme included TB drug resistance survey in its strategy. The effort to establish laboratory capacity for culture and TB drug susceptibility testing was delayed by compounded challenges as the programme didn’t put effective strategy to establish standard diagnosis and treatment mechanism for MDR-TB. Liberia is one of the few countries in the world where national TB prevalence survey has never been conducted and the accurate national TB burden is not yet established. The country must adopt and implement the guidelines and standards developed by World Health Organization to address diagnosis, treatment and infection control of TB.

Despite policies, strategies and guidelines, the epidemic of HIV-associated tuberculosis continues to rage, particularly in Africa [26]. Review of the NLTCP documents and registers indicated that the percentage of HIV+ TB patients with known HIV test result was 11%, 49%, 55% and 55% from the year 2009 to 2012 respectively. The rapid expansion of HIV testing for TB patients has been particularly encouraging in Africa where only 4% of TB patients were tested for HIV in 2004 but by 2008 that percentage had increased to 45% [27].

The percentage of HIV + TB patients started or continued on co-trimoxazole preventive therapy was 42% in 2012 and reached 74% in 2013. In similar review of the NLTCP documents, the percentage of HIV+ TB patients started or continued on Anti-retroviral therapy (ART) was 6% in 2010 and reached 16% in 2013. In this study out of 139 TB patients that participated, one hundred thirty (94%) had an HIV test result known, 40 (67%) of the HIV positive participants were ART initiated whereas 20 (33%) were not initiated. The data review of this study indicated that the recording of TB/HIV collaborative activities was found to be inconsistent in a number of facilities. These include discrepancies between what was on the quarterly report and on the TB registers. Shortage of HIV testing kits was also noted during the discussion and interview with the study participants. To fill the gaps identified in this study, both HIV and tuberculosis programmes have to exhort implementation strategies that are known to be effective and also set innovative strategies that could work. The continuing HIV-associated tuberculosis epidemic needs coordinated and sustainable action by the NLTCP and National Aids Control Programme of Liberia. The NLTCP of Liberia has critical gaps in the implementation of the three Donabedian’s components.

### Demographic, patient and socio-economic factors that determine the performance of TB programme of Liberia from patients’ perspectives

Patient related demographic, socio-economic, disease and medicine related factors were examined to evaluate the performance of TB programme of Liberia. To explore patients’ perspectives TB treatment compliance was used as one part of this study. Treatment compliance is a complex and dynamic phenomenon with a wide range of interacting factors impacting treatment taking behaviour. It poses a significant threat to both the individual patient and public health and is associated with higher transmission rates, morbidity and costs of TB control programmes [28]. The socio-demographic factors included age, gender, marital status, level of formal education and religion. A total of 139 participants were interviewed of which 120 completed TB treatment and 19 did not complete TB treatment. Age, marital status, religion and education level attained did not contributed to any significant difference between the treatment compliant and non-compliant groups (Table 1). The only demographic factor that significantly contributed to difference in compliance was gender. More males were found in the non-compliant group than females. A study conducted in Nigeria [29] reported that male sex was a good predictor of poor treatment outcome. The reason for these could be attributed to the fact that in most societies, men are the bread winners in the family and they tend to leave home early for work in order to provide income for their families and thus find it difficult to comply with daily TB clinic attendance.

Smoking, drinking alcohol and availability of a treatment supporter were patient-related factors included in this study. A total of 10 (7%) of the participants reported having smoked cigarettes during the previous six months while 129 (93%) had not done so. Smoking cigarettes in the previous six months on TB treatment was however not associated with any statistical difference between the compliant and non-compliant groups. In contrary, a study conducted by Bagchi and Sathiakumar [30], smoking during treatment was significantly associated with non-adherence to treatment.

Poor socio-economic status is associated with risks of tuberculosis infection, dissemination and with inadequate and delayed availability of health care. Poverty also results in poor nutrition and low body weight which are likely to render the immune system more vulnerable to the invading organisms [31]. The socio-economic factors described in this study included the employment status, level of income and availability of food during the period the patient was on TB treatment. In this study, of the 139 interviewed, only 30 (21%) were employed, 76 (54%) were unemployed and 33 (24%) reported being self-employed. Participants who were unemployed were more non-compliant compared to the employed and the self- employed. Unemployment was a significant factor contributing non-compliance to TB treatment. This is similar to the findings in some Sub-Saharan African countries socioeconomic factors such as low income and low education which were linked to TB treatment non-compliance [32]. Regarding food availability, in this study, only 53 (38%) of the participants reported that food was always available to take with their medicines, 24 (17%) said food was not always available and for 58 (42%), food was available most of the time. Four (3%) said food was never available to take with their medicine. Availability of food was a significant factor affecting TB treatment compliance in this study. The findings are similar with the studies conducted in Kenya and Pakistan where TB patients cited lack of food as a reason for discontinuing treatment [17, 33].

The other determinant factors included in this study were housing condition, number of people living in the current house and length of stay in the current house. In this study; a higher proportion of patients in the non-compliant group had stayed in their present residence for less than twelve months, had fewer rooms and a higher number of people living in the same house, though there was no significant difference between the compliant and non-compliant groups. This implies that patients with less stable living conditions did not comply with treatments. Crowding, poor air quality within homes as a result of inadequate ventilation and the presence of mold and smoke contribute to poor respiratory health in general and have been implicated in the spread and poor outcome of tuberculosis [34].

Stigma affects the quality of patients’ lives and the effectiveness of TB control. The finding of this study indicated that 84 (60%) of the participants had disclosed their TB status to either a family member or friend and 55 (40%) didn’t disclose their TB status. Those who disclosed their TB status were more compliant compared to those who didn’t. A study conducted in Ghana indicated that most TB patients failed to state that their symptoms were due to TB, because of the stigma attached to the disease in society [19]. Studies at four sites; Bangladesh, India, Malawi and Colombia have identified common features of TB-related stigma that seriously affected the TB treatment compliance [35].

## Conclusion

The finding of this study indicated that the National Leprosy and Tuberculosis Control Programme of Liberia didn’t meet the Millennium Development Goals and Stop TB Partnership targets of 100% DOTS coverage, 70% case detection rate of all forms of TB and treatment success rate of above 85%. The lack of adequate government funding crippled the programme and the programme was not performing to meet the global targets as well as the programme targets. The three Donabedian’s components were used to assess the overall performance of the NLTCP of Liberia. Lack of adequate financial resources, separate laboratories and under staffing at health facilities which provide TB services were some of the gaps identified from the structure aspect of Donabedian’s model. Inadequate information about TB from the health workers, long patient waiting time to receive services at each of the levels of care, poor attitude of fellow health workers was some of the key Donabedian’s process issues hindering TB control in Liberia. Limitation in laboratory reagents slides for sputum microscopy and TB laboratory SOPs; infrequent health education for attendants and absence of sputum quality control procedures and mechanism to trace defaulters and shortage of drugs were some of the bottle necks identified in this study. The Donabedian’s treatment outcome factors of case detection and treatment success rate are not met. The high default rate of this study indicates that the programme has to put effective strategies of defaulter tracing and patient centered treatment approaches for tuberculosis as part of promoting approaches that tackle underlying roots of stigma. Comprehensive strengthening of the health system focusing on quality of support supervisions, patient follow up and promoting infection control measures and increasing health staffing levels at the health facilities are crucial. The National Leprosy and Tuberculosis Control Programme of Liberia have to mobilise resources and ensure sustainable budget to improve the performance of the programme and meet global targets.

## Supporting information

S1 Table Patient demographic, socio-economic and health related data. (XLS)

S2 Table Tuberculosis Laboratory proficiency panel performance data. (XLS)

S3 Table NLTCP of Liberia Program performance data. (XLSX)

S4 Table Program performance treatment outcome data. (XLS)

S1 Text Study participants’ consent form. (PDF)

## References

[1] Harries AD & Dye C. Tuberculosis. Annals of Tropical Medicine and Parasitology. 2006; 100(5–6):415–431. doi: 10.1179/136485906X91477 16899146 10.1179/136485906X91477

[2] Gerald L, John E & Raphael B. Principles and practice of infectious diseases. 7th edition:1–3129; Philadelphia, PA: Churchill Livingstone/Elsevier 2010.

[3] World Health Organization. Tuberculosis global report Geneva: WHO. Press 2012b.

[4] Lawn SD & Zumla Al. Tuberculosis. Lancet. 2011; 378(9785):57–72. doi: 10.1016/S0140-6736(10)62173-3 21420161 10.1016/S0140-6736(10)62173-3

[5] World Health Organization. The global plan to stop TB, 2006–2015: Action for life: Towards a world free of Tuberculosis. Geneva, Switzerland: WHO Press 2006.

[6] Leprosy National & TB Control Programme. *Manual of the National Tuberculosis and Leprosy Program in Liberia*. MOH & SW, Liberia. 2009;1–5.

[7] Donabedian A. An introduction to quality assurance in health care New York: Oxford University Press 2003: 1–240.

[8] Patrick K, Gorgui D, Augustine T, Moses T & Shaofa N. A Retrospective Study to Determine the Prevalence and Outcome of Tuberculosis among Patients Who Visited the TB Annex Hospital in Congo Town, Monrovia, Liberia from July 2009 to July 2010. Global Journal of Health Science. 2011; 3(1):110–118.

[9] Mays N & Pope C. Qualitative research in health care: Assessing quality in qualitative research. British Medical Journal. 2000; 320:50–52. 10617534 10.1136/bmj.320.7226.50 PMC1117321

[10] LoBiondo-Wood, G & Haber, J. *Nursing research: Methods, critical appraisal and utilization*. 2005; 6th edition: 277–279. 10.7748/ns.4.38.6.s57 27238519

[11] Bruce N, Pope D & Stanistreet D. Quantitative methods for health research. Division of Public Health, University of Liverpool, UK 2008: 1–538.

[12] Graneheim UH & Lundman B. Qualitative content analysis in nursing research: Concepts, procedures and methods to achieve trustworthiness. Nurse Education Today. 2004; 24:105–112. doi: 10.1016/j.nedt.2003.10.001 14769454 10.1016/j.nedt.2003.10.001

[13] Amo-Adjei J. Political commitment to tuberculosis control in Ghana. Global Public Health. 2014; 9(3):299–311. doi: 10.1080/17441692.2014.880500 24521048 10.1080/17441692.2014.880500

[14] Addo K, Dan-Dzide M, Yeboah-Manu D & Owusu-Darko K. Improving the laboratory diagnosis of TB in Ghana: the impact of a quality assurance system. International Journal of Tuberculosis and Lung Disease. 2006; 10(7):812–817. 16848346

[15] Salla A, Munro A, Lewin E, Atle F & Jimmy V. Patient adherence to Tuberculosis treatment: A systematic review of qualitative research. PLoS Medicine. 2007; 4(7):1230–1245. 10.1371/journal.pmed.0040238 PMC1925126 17676945

[16] Harper M, Ahmadu FA, Ogden JA, McAdam KP & Lienhardt C. Identifying the determinants of tuberculosis control in resource-poor countries: Insights from a qualitative study in Gambia. Transaction of the Royal Society of Tropical Medicine and Hygiene. 2003; 97:506–510. 10.1016/s0035-9203(03)80007-x 15307411

[17] Mature B, Keraka M, Kimmu P, Kabiru E, Ombeka V & Oguya F. Factors associated with default from treatment among tuberculosis patients in Nairobi province, Kenya. A case control study. *BMC Public Health*. 2011; 11:6–96. 10.1186/1471-2458-11-696 PMC3224095 21906291

[18] Khan MA, Walley JD, Witter SN, Shah SK & Javeed S. Tuberculosis patient adherence to direct observation: Results of a social study in Pakistan. Health Policy Plan. 2005; 20:354–365. doi: 10.1093/heapol/czi047 16183735 10.1093/heapol/czi047

[19] Dodor EA.The feelings and experiences of patients with Tuberculosis in the Sekondi-takoradi metropolitan district: implications for TB control efforts. Ghana Medical Journal. 2012; 46(4): 211–218. 23661839 PMC3645176

[20] James M, M’imunya T & Volmink J. Patient education and counselling for promoting adherence to treatment for tuberculosis Cochrane Database of Systematic Reviews. Published by John Wiley & Sons 2012; 1–31. 10.1002/14651858.CD006591.pub2 PMC6532681 22592714

[21] Bulage L, Sekandi J, Kigenyi O& Mupere E. The Quality of Tuberculosis services in health care centres in a rural district in Uganda: The providers’ and clients’ perspective. Hindawi Publishing Corporation Tuberculosis Research and Treatment. 2014:1–11. 10.1155/2014/685982 PMC4170836 25276424

[22] Daniel OJ, Oladapo OT & Alausa OK. Default from treatment program in Sagamu, Nigeria. Nigeria Journal of Medicine. 2006; 15(1):63–67. 10.4314/njm.v15i1.37119 16649455

[23] Wasonga J. Factors contributing to tuberculosis treatment defaulting among slum dwellers in Nairobi, Kenya, International congress on drug therapy in HIV Kenya: The Gardiner-Caldwell Group 2006; 310.

[24] Mauch V, Weil D, Munim A, Boillot F, Coninx R, Huseynova S, Powell C, Seita A, Wembanyama H and Van den S. Structure and management of tuberculosis control programmes in fragile states: Afghanistan, DR Congo, Haiti, Somalia. Health policy. *Netherlands*. 2010; 96(2):118–27. doi: 10.1016/j.healthpol.2010.01.003 20170977 10.1016/j.healthpol.2010.01.003

[25] Ahmed M, Sodemann M & Aro A. Evaluation of tuberculosis control programme in Khartoum State for the year 2006. Scandinavian Journal of Public Health. 2009; 37(1):101 doi: 10.1177/1403494808097172 19141559 10.1177/1403494808097172

[26] Harries AD, Zachariah R, Corbett EL, Lawn SD, Santos ET, Chimzizi R, Harrington M, Maher D, Williams BG, Cock KM. The HIV-associated tuberculosis epidemic–when will we act? The Lancet. 2010; 375(9729):1906–9. 10.1016/S0140-6736(10)60409-6 20488516

[27] Padmapriyadarsini C, Narendran G & Swaminathan S. Diagnosis and treatment of tuberculosis in HIV co-infected patients. Indian Journal of Medical Research. 2011; 134(6):850–865. doi: 10.4103/0971-5916.92630 22310818 10.4103/0971-5916.92630 PMC3284094

[28] Mubashir Z. Initiation and adherence to TB treatment in a Pakistani community influenced more by perceptions than by knowledge of tuberculosis. *The Journal of Association of Chest Physicians* (JACP). 2013; 2(1):45.

[29] Inotu A & Abebe F. Assessment of Defaulting from Directly Observed Treatment Short Course (DOTS) and its determinants in Benin City, Nigeria. Journal of Tuberculosis Research. 2014; 2:30–39.

[30] Bagchi S, Ambe G & Sathiakumar N. Determinants of poor adherence to anti-tuberculosis treatment in Mumbai, India. International Journal of Preventive Medicine 2010; 1(4):1–22. 21566777 PMC3075517

[31] Dheeraj G, Kshaunish D, Balamughesh T, Ashutosh N. & Surinder K. Role of socio-economic factors in tuberculosis prevalence. Indian Journal Tuberculosis. 2004; 51:27–31.

[32] Dodor EA & Afenyandu GY. Factors associated with tuberculosis treatment default and completion at Effia-Nkwanta Regional Hospital in Ghana. Transactions of the Royal Society of Tropical Medicine and Hygiene. 2005; 99(11):827–832. doi: 10.1016/j.trstmh.2005.06.011 16102791 10.1016/j.trstmh.2005.06.011

[33] Padmanesan N, James W, Chandini R & Dilip M. Risk factors for Tuberculosis. Pulmonary Medicine. 2013; 1–11.23305075

[34] Wanyeki I, Olson S & Brassard P. Dwellings, crowding, and tuberculosis in Montreal. Social Science and Medicine. 2006; 63:501–511. doi: 10.1016/j.socscimed.2005.12.015 16480805 10.1016/j.socscimed.2005.12.015

[35] Gosoniu GD, Ganapathy S & Kemp J. Cultural epidemiological determinants of delay to diagnosis of TB in Bangladesh, India and Malawi. International journal of Tuberculosis and Lung Disease. 2008; 12:848–855 18544215

