# Supplementary material for: Performance of the National Tuberculosis Control Program in the post conflict Liberia
Source: PLoS One. 2018 Jun 25;13(6):e0199474. doi: 10.1371/journal.pone.0199474 (PMC6016901; doi:10.1371/journal.pone.0199474)
Supplement: S1 Text — (PDF) [file pone.0199474.s005.pdf]

Study participants' consent

Dear Sir/Madam

You are requested to participate in a study about National Tuberculosis Program performance in which factors that contribute to the Tuberculosis treatment compliance by Tuberculosis patients are also investigated. The findings of the study will be used to improve TB patient care management and thus reduce further spread of TB in the community. Your participation or your non- participation will in no way affect you or your family regarding the services you require from the facility. There is no direct benefit that you will receive by participating in the study, but you will contribute towards the improvement of the Tuberculosis services after that the needs for TB patients have been identified.

Your participation in this study is voluntary. I therefore request you to assist with answering the questions included in this questionnaire. The information you may give us today could help us achieve this. Please note that any information which may identify you will be kept strictly confidential and your responses will in no way lead to any adverse effect on you and no medical care will be withheld from you because of the responses you may provide. If you agree to this interview, you may sign below but if you do not agree, you can let me know at this point and I will not proceed with the interview.

For any information regarding the study, you may contact Kassaye Tekie Desta; Telephone number +231886929898.

DATE:______________________________

PARTICIPATE'S SIGNATURE: _________________________
